# ETHE1 and MOCS1 deficiencies: Disruption of mitochondrial bioenergetics, dynamics, redox homeostasis and endoplasmic reticulum-mitochondria crosstalk in patient fibroblasts

**DOI:** 10.1038/s41598-019-49014-2

**Published:** 2019-09-02

**Authors:** Mateus Grings, Bianca Seminotti, Anuradha Karunanidhi, Lina Ghaloul-Gonzalez, Al-Walid Mohsen, Peter Wipf, Johan Palmfeldt, Jerry Vockley, Guilhian Leipnitz

**Affiliations:** 1 Programa de Pós-Graduação em Ciências Biológicas: Bioquímica, Universidade Federal do Rio Grande do Sul, Rua Ramiro Barcelos, 2600-Anexo, CEP 90035-003 Porto Alegre, RS Brazil; 2 Division of Medical Genetics, Department of Pediatrics, University of Pittsburgh, Pittsburgh, PA 15224 USA; 3 Department of Human Genetics, Graduate School of Public Health, University of Pittsburgh, Pittsburgh, PA 15213 USA; 4 Departments of Chemistry, Pharmaceutical Sciences and Bioengineering, University of Pittsburgh, Pittsburgh, PA 15260 USA; 5 Research Unit for Molecular Medicine, Aarhus University Hospital, Skejby, Denmark; 6 Departamento de Bioquímica, Instituto de Ciências Básicas da Saúde, Universidade Federal do Rio Grande do Sul, Rua Ramiro Barcelos, 2600-Anexo, CEP 90035-003 Porto Alegre, RS Brazil

**Keywords:** Biochemistry, Biochemistry, Clinical genetics, Clinical genetics

## Abstract

Ethylmalonic encephalopathy protein 1 (ETHE1) and molybdenum cofactor (MoCo) deficiencies are hereditary disorders that affect the catabolism of sulfur-containing amino acids. ETHE1 deficiency is caused by mutations in the *ETHE1* gene, while MoCo deficiency is due to mutations in one of three genes involved in MoCo biosynthesis (*MOCS1*, *MOCS2 and GPHN*). Patients with both disorders exhibit abnormalities of the mitochondrial respiratory chain, among other biochemical findings. However, the pathophysiology of the defects has not been elucidated. To characterize cellular derangements, mitochondrial bioenergetics, dynamics, endoplasmic reticulum (ER)-mitochondria communication, superoxide production and apoptosis were evaluated in fibroblasts from four patients with ETHE1 deficiency and one with MOCS1 deficiency. The effect of JP4-039, a promising mitochondrial-targeted antioxidant, was also tested on cells. Our data show that mitochondrial respiration was decreased in all patient cell lines. ATP depletion and increased mitochondrial mass was identified in the same cells, while variable alterations in mitochondrial fusion and fission were seen. High superoxide levels were found in all cells and were decreased by treatment with JP4-039, while the respiratory chain activity was increased by this antioxidant in cells in which it was impaired. The content of VDAC1 and IP3R, proteins involved in ER-mitochondria communication, was decreased, while DDIT3, a marker of ER stress, and apoptosis were increased in all cell lines. These data demonstrate that previously unrecognized broad disturbances of cellular function are involved in the pathophysiology of ETHE1 and MOCS1 deficiencies, and that reduction of mitochondrial superoxide by JP4-039 is a promising strategy for adjuvant therapy of these disorders.

## Introduction

The breakdown of the sulfur-containing amino acids methionine and cysteine occurs in the cytosol of cells and leads to the formation of hydrogen sulfide (H_2_S) and sulfite, which are further degraded by mitochondrial enzymes^1^. H_2_S is metabolized through a pathway including ethylmalonic encephalopathy protein 1 (ETHE1), a sulfur dioxygenase found in the mitochondrial matrix that also plays a fundamental role in the elimination of H_2_S produced by gut anaerobic bacteria^1,2^. Sulfite is oxidized to sulfate by sulfite oxidase (SO), an enzyme located in the mitochondrial intermembrane space that depends on a molybdenum cofactor (MoCo) as an essential cofactor^3,4^. SO is also important for the detoxification of sulfites used as preservatives in foods and medicines^5–7^. Mutations in the genes encoding ETHE1, SO and the enzymes of the MoCo biosynthetic pathway lead to disorders characterized by the accumulation of toxic compounds, and severe and progressive neurological manifestations^8,9^.

Patients with ETHE1 deficiency, also known as ethylmalonic encephalopathy, present with early onset progressive neurological degeneration, psychomotor retardation, microvasculature injury consisting of orthostatic acrocyanosis and petechial purpura, and chronic diarrhea, resulting usually in death in the first few years of life^10,11^. Brain hemorrhagic lesions, bilateral necrosis in the basal ganglia and brainstem, and abnormalities in the cerebral cortex, cerebellum and corpus callosum are some of the main neuropathological and neuroimaging findings^2,10–12^. High levels of H_2_S and thiosulfate are observed in biological fluids and tissues, as well as high plasma levels of lactate, short chain acylcarnitines and ethylmalonic acid, with markedly elevated urinary excretion of methylsuccinic acid^10^. Patient tissues and cells exhibit an impaired mitochondrial respiratory chain function and inhibition of the short-chain acyl-CoA dehydrogenase by H_2_S^13–15^.

SO deficiency may arise from mutations in the *SUOX* gene, that encodes SO (isolated SO deficiency – ISOD), or the enzymes involved in the biosynthesis of the MoCo (molybdenum cofactor deficiency – MoCD)^16^. Mutations in *MOCS1*, *MOCS2* and *GPHN* result in MoCD type A, B and C, respectively^16^. MoCDs are clinically characterized by severe neonatal seizures and progressive encephalopathy, often leading to death in early childhood. Other symptoms may include axial hypotonia with peripheral hypertonicity, failure to thrive, and ectopia lentis^8,17^. Neuroimaging studies show multicystic lesions with marked neuronal loss, gliosis, and demyelination, as well as cerebral cortex, basal ganglia, cerebellum, thalamus and corpus callosum atrophy^18–20^. Increased lactate levels in brain structures are also observed^21–24^. MoCD leads to accumulation of sulfite, thiosulfate and S-sulfocysteine in tissues and biological fluids. Low uric acid and high xanthine and hypoxanthine levels are also found, as the xanthine oxidase and aldehyde oxidase are also dependent on MoCo^8,21,25^.

The pathophysiological mechanisms responsible for the symptoms observed in patients with ETHE1 deficiency and MoCD have not been fully established. However, studies conducted in rodents have shown that the metabolites accumulated in these disorders, mainly H_2_S and sulfite, exert toxic effects, inducing oxidative stress and impairment of cellular energy metabolism^15,26–29^. Moreover, studies with *Ethe1*^−/−^ mice and patient fibroblasts strengthen the concept that disturbances in redox homeostasis are involved in ETHE1 deficiency^13,30,31^.

Treatment options for these disorders are limited. Cyclic pyranopterin monophosphate (cPMP), an intermediate in the MoCo biosynthetic pathway, has been used to treat patients with MoCD type A, but requires institution in a time frame not realistic in the context of new patients^32,33^. This compound is not effective for ISOD and MoCD type B and C, and dietary therapy with reduced content of sulfur-containing amino acids has had minimal clinical success^8,34–36^. In patients with ETHE1 deficiency, a combination of N-acetylcysteine (a precursor of the H_2_S scavenger glutathione) and metronidazole (an antibiotic that decreases anaerobic bacteria in the gut) have been reported to have benefits, though it was not effective in all cases^13,37^. Therefore, further studies evaluating potential protective compounds are of high interest in order to develop new therapeutic approaches and improve the prognosis of these disorders.

JP4-039 is a novel synthetic mitochondrial-targeted antioxidant composed of a nitroxide group with reactive oxygen species (ROS) and electron scavenger properties, and a portion derived from the antibiotic gramicidin S, which is responsible for its selective accumulation in mitochondria^38,39^. JP4-039 has been shown to attenuate radiation damage, prevent lipoperoxidation and apoptosis in animal models and different tumor cell lines, as well as to improve mitochondrial respiration and scavenge ROS in complex I deficient fibroblasts^40^.

The present work investigated the pathophysiology of ETHE1 deficiency and MoCD, assessing ROS production, mitochondrial bioenergetics and dynamics, endoplasmic reticulum (ER)-mitochondrial communication, ER stress and apoptosis in fibroblasts of patients diagnosed with these diseases. Additionally, the effect of JP4-039 on ROS generation and mitochondrial function was evaluated.

## Methods

Methods were performed in accordance with the approved guidelines and regulations. Experimental human protocols were approved by the Institutional Review Board at the University of Pittsburgh, protocol IRB0404017.

### Subjects

One fibroblast cell line with mutations in the gene *MOCS1* (MoCD type A) was obtained from *Coriell Research Institute for Medical Research* (Camden, NJ, USA), and four fibroblast cell lines with mutations in the gene *ETHE1* (from patients with ETHE1 deficiency) were provided by Prof. Johan Palmfeldt (Aarhus University Hospital, Denmark). Skin biopsies from patients were performed on a clinical basis with written informed consent from patients and/or parents. Banked fibroblasts from healthy individuals were used as controls (wild type cells).

### Cell culture and treatments

Cells were routinely grown in Dulbecco’s Modified Eagle Medium (DMEM), Corning Life Sciences, Manassas, VA, USA, containing high glucose levels (4.5 g/L) and supplemented with 10% fetal bovine serum, 4 mM glutamine, 100 IU penicillin and 100 μg/mL streptomycin, Corning Life Sciences, Manassas, VA, USA, at 37 °C, 5% (v/v) CO_2_. For some experiments, fibroblasts were pre-treated for 24 h or 7 days with JP4-039 (40 or 200 nM), obtained from Dr. Peter Wipf, Department of Chemistry, University of Pittsburgh, USA^41,42^ or *N*-acetylcysteine (0.5 or 1 mM), Sigma-Aldrich Co., St. Louis, MO, USA. Cells were used at a passage number not greater than 14.

### Mutation analysis

Genomic DNA from fibroblasts was extracted using a *Quick*-DNA^TM^ Miniprep Plus Kit, Zymo Research, Irvine, CA, USA, according to manufacturer’s instructions, followed by PCR to amplify desired regions on each gene. Gel extraction of the PCR product was performed with a Zymoclean^TM^ Gel DNA recovery kit, Zymo Research, followed by Sanger sequencing.

Total RNA was isolated from fibroblasts using a Quick-RNA^TM^ MiniPrep kit, Zymo Research, and cDNA was synthesized using SuperScript III First-Strand Synthesis System, Thermo Fisher Scientific, Waltham, MA, USA, according to manufacturer’s instructions. cDNA was used for RT-PCR, and gel extraction of the PCR products was performed with the Zymoclean^TM^ Gel DNA recovery kit, Zymo Research, followed by Sanger sequencing.

### Mitochondrial respiration

The oxygen consumption rate (OCR) was measured with a Seahorse XF^e^96 Extracellular Flux Analyzer, Agilent Technologies, Lexington, MA, USA. Cells were seeded in 96-well Seahorse tissue culture microplates pre-coated with poly-D-lysine (80,000 cells per well), Sigma-Aldrich Co., St. Louis, MO, USA, for cell adherence. Subsequently, the plate was incubated, prior to the assay, at 37 °C for 1 h without CO_2_ in basal media containing unbuffered DMEM. The initial OCR was evaluated to establish the basal respiration, the ATP-linked respiration was determined following injection of the complex V inhibitor oligomycin and the maximal respiration following the injection of 1 µM of the uncoupling agent FCCP. All cell lines were measured with four to eight wells per cell line. After the assay, protein concentration in each well was measured using the *DC*™ Protein Assay kit, Bio-Rad Laboratories, Hercules, CA, USA. Data were expressed in pmol/min/mg of protein.

### ATP production

ATP production was measured by bioluminescence using an ATP determination kit (ATPlite™), PerkinElmer Inc., Waltham, MA, USA, according to the manufacturer’s instructions. Luminescence was quantitated in a SpectraMax® i3x Platform multi-mode microplate reader system, Molecular Devices LLC, Sunnyvale, CA, USA. After the assay, the protein concentration in each well was measured using the *DC*™ Protein Assay kit, Bio-Rad Laboratories, Hercules, CA, USA. Data are reported in μmol of ATP produced/mg of protein.

### Mitochondrial mass and superoxide production

Mitochondrial mass and superoxide production were measured using the probes MitoTracker^®^ Green and MitoSOX^®^ Red, Invitrogen, Carlsbad, CA, USA, respectively. A cell suspension containing 1 × 10^5^ cells per mL of DMEM was incubated concomitantly with 150 nM MitoTracker^®^ Green and 5 µM MitoSOX^®^ Red for 20 min at 37 °C. After incubation, 10,000 cells per sample were evaluated in a Becton Dickinson FACSAria II flow cytometer, BD Biosciences, San Jose, CA, USA. Data were expressed as fluorescent arbitrary units (FAU).

### Western blotting

Cells were grown in T175 flasks and, at 95–100% confluence, harvested by trypsinization, pelleted and stored at −80 °C. For whole cell lysate preparation, the pellets were resuspended in RIPA buffer containing a protease inhibitor cocktail, Roche Diagnostics, Mannheim, Germany. Homogenates were kept on ice for 30 min and mixed every 10 min, then centrifuged at 14,000 *g* for 10 min at 4 °C, and the supernatant was collected. Protein content was determined using the *DC*™ Protein Assay kit, Bio-Rad Laboratories, Hercules, CA, USA, and equal amounts of protein (30 μg) of each sample were treated with Laemmli-sample buffer (62.5 mM Tris HCl, pH 6.8, 1% (w/v) SDS, 10% (v/v) glycerol), fractioned by SDS-PAGE and blotted onto nitrocellulose membranes. The membranes were then blocked with 5% skimmed milk or 5% bovine serum albumin for 1 h and washed with Tris-buffered saline and Tween 20 (TBST; Tris 100 mM, pH 7.5, 0.9% NaCl and 0.1% Tween-20). Overnight incubation of the membranes at 4 °C with primary antibody was then performed. The primary antibodies mouse anti-MOCS1 monoclonal antibody (1:100), mouse anti-MFN2 monoclonal antibody (1:300) and goat anti-IP3R polyclonal antibody (1:50) were purchased from Santa Cruz Biotechnology, Dallas, TX, USA, the antibodies mouse anti-SO polyclonal antibody (1:500), mouse anti-ETHE1 polyclonal antibody (1:500), mouse anti-MFN1 monoclonal antibody (1:500), mouse anti-DRP1 monoclonal antibody (1:500), mouse anti-OPA1 monoclonal antibody (1:1000), rabbit anti-VDAC1 monoclonal antibody (1:1,000), mouse anti-Grp75 monoclonal antibody (1:250), rabbit anti-Grp78 polyclonal antibody (1:250), mouse anti-DDIT3 monoclonal antibody (1:250) were from Abcam, Cambridge, MA, USA, and the antibodies rabbit anti-phospho DRP1 (Ser616) polyclonal antibody (1:500), and rabbit anti-phospho DRP1 (Ser 637) polyclonal antibody (1:500) were from Cell Signaling Technology, Danvers, MA, USA. After incubation, membranes were washed with TBST and incubated with anti-mouse or rabbit IGg HRP-conjugated secondary antibody for 1 h at room temperature. The membranes were washed again with TBST and immersed in chemiluminescence (ECL) detecting substrate, Millipore, Billerica, MA, USA, for 2 min, and revealed with an image system. Protein bands were quantitatively analyzed with the NIH ImageJ Analysis software and normalized to a loading control (HRP-conjugated anti-β-actin monoclonal antibody; 1:62,500; Proteintech, Rosemont, IL, USA). Gel images from all figures conform to the journal policy as noted at www.nature.com/srep/policies/index.html#digital-image. Images were electronically adjusted to optimize comparisons within a single gel. High contrast and overexposed images were not utilized.

### Apoptosis assay

Apoptosis was evaluated with an Alexa Fluor® 488 annexin V/Dead Cell Apoptosis kit, Invitrogen, Carlsbad, CA, USA, according to manufacturer’s instructions. The kit contains annexin V labeled with a fluorophore and PI. Annexin V can identify apoptotic cells by binding to phosphatidylserine exposed on the outer leaflet of cell plasma membrane while PI stains dead cells by binding to nucleic acids. Fluorescence was determined in a Becton Dickinson FACSAria II flow cytometer, BD Biosciences. Data were expressed as fluorescent arbitrary units (FAU).

### Statistical analysis

Statistical analysis was performed with GraphPad 5.0 software. Student’s *t* test or one-way ANOVA followed by Tukey multiple range test were applied for comparisons between groups. Differences were considered significant when *P* < 0.05.

## Results

### Mutation analysis and protein content of *ETHE1*, *MOCS1* and *SO*

**Figure 1:**
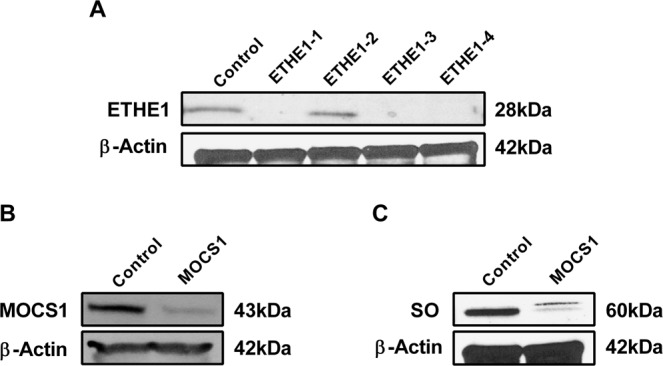
Decreased levels of ETHE1, MOCS1 and SO protein. Protein content of ETHE1 (**A**) MOCS1 (**B**) and SO (**C**) was measured in whole cell lysates prepared from ETHE1 and MOCS1 deficient cells. β-actin was used as a loading control.

Sequencing of *ETHE1*, *MOCS1*, *MOCS2* and *MOCS3* from genomic DNA and cDNA was performed to confirm the previously reported mutations of fibroblasts from four patients with ETHE1 deficiency^30^ and determine the mutation of a fibroblast cell line with MoCD (Supplementary Information: Table S1). Fibroblast levels of ETHE1 and MOCS1 (molybdenum cofactor biosynthesis protein 1; MOCS1A and MOCS1B; 2 enzymes encoded by *MOCS1*) proteins were then evaluated by western blotting (Fig. 1). Cell lines ETHE1-1, ETHE1-3 and ETHE1-4 had no immunodetectable ETHE1 protein (cell lines with exon deletion or splice site mutation) while ETHE1-2 had similar ETHE1 protein as control cells, indicating that the missense mutation in ETHE1-2 leading to the substitution of Asp165 by a Gly causes complete loss of ETHE1 activity. MOCS1 deficient cells had a reduced amount of MOCS1 (MOCS1A and MOCS1B), as well as SO.

### Oxygen consumption and ATP production

**Figure 2:**
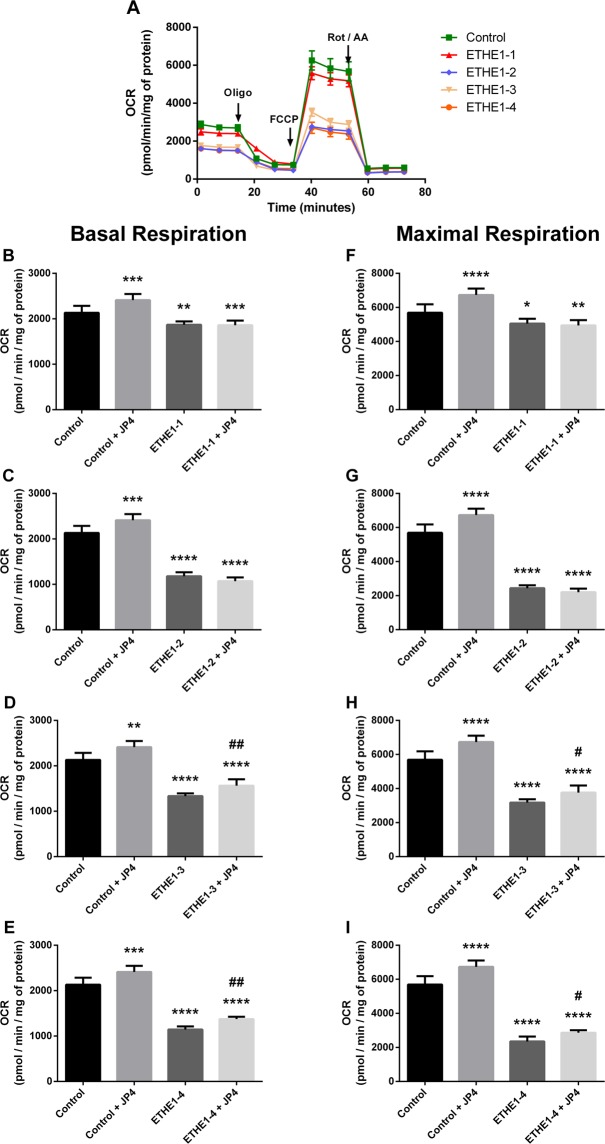
Mitochondrial respiration impairment in ETHE1 deficient fibroblasts. Oxygen consumption rate (OCR) was measured in the resting state (basal respiration) followed by injection of oligomycin (an inhibitor of ATP synthase) that reduces OCR, representing ATP turnover. Subsequent injection of FCCP dissipates the proton gradient and allows maximum respiration. Finally, rotenone (Rot) and antimycin A (AA) are added to completely disable the electron transport chain, inhibiting the total mitochondrial respiration. The remaining OCR represents non-mitochondrial respiration (**A**). Basal respiration of ETHE1-1 (**B**), ETHE1-2 (**C**), ETHE1-3 (**D**) and ETHE1-4 fibroblasts (**E**), and maximal respiration of ETHE1-1 (**F**), ETHE1-2 (**G**), ETHE1-3 (**H**) and ETHE1-4 (**I**) fibroblasts exposed or not to 40 nM JP4-039 (JP4) for 24 h. Data are means ± SD; number of replicates: 7–8. *P < 0.05, **P < 0.01, ***P < 0.001, ****P < 0.0001, compared to control (wild type) cells; ^#^*P* < 0.05, ^##^*P* < 0.01 compared to patient cells (Tukey multiple range test).

**Figure 3:**
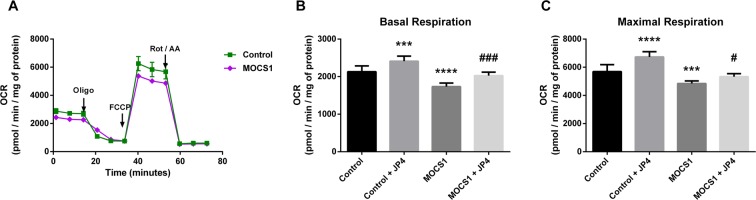
Mitochondrial respiration impairment in MOCS1 deficient fibroblasts. OCR (**A**) was measured as detailed in the legend for Fig. 2. Basal respiration (**B**) and maximal respiration (**C**) of MOCS1 deficient fibroblasts treated or not with 40 nM JP4-039 (JP4) for 24 h. Data are means ± SD; number of replicates: 7–8. ***P < 0.001, ****P < 0.0001, compared to control (wild type) cells; ^#^*P* < 0.05, ^###^*P* < 0.001 compared to MOCS1 deficient cells (Tukey multiple range test).

**Figure 4:**
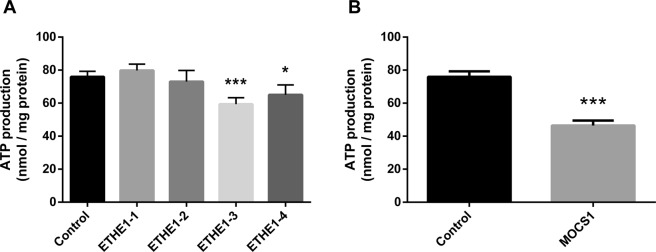
Decreased ATP levels in ETHE1 (**A**) and MOCS1 (**B**) deficient fibroblasts. Data are presented as mean ± SD; number of replicates: 5. *P < 0.05, ***P < 0.001, as compared to control (wild type) cells (Tukey multiple range test and *t* test for unpaired samples).

As a measure of the bioenergetic state of patient fibroblasts oxygen consumption was determined using a Seahorse analyzer. Basal, maximal and ATP-linked respiration was decreased in all cell lines (Figs 2, 3 and Supplementary Fig. S1). Treatment of cells with 40 nM JP4-039 for 24 h increased these measurements in ETHE1-3 and ETHE1-4 (Fig. 2) and MOCS1 deficient cells (Fig. 3). ATP production measured with ATPlite luminescence assay kit was also decreased in ETHE1-3 and ETHE1-4 and the MOCS1 deficient cells (Fig. 4). No alterations were observed in ETHE1-1 and ETHE1-2 cell lines. Taken together, these data show bioenergetic impairment in MOCS1 and some ETHE1 deficient fibroblasts.

### Mitochondrial mass and dynamics

**Figure 5:**
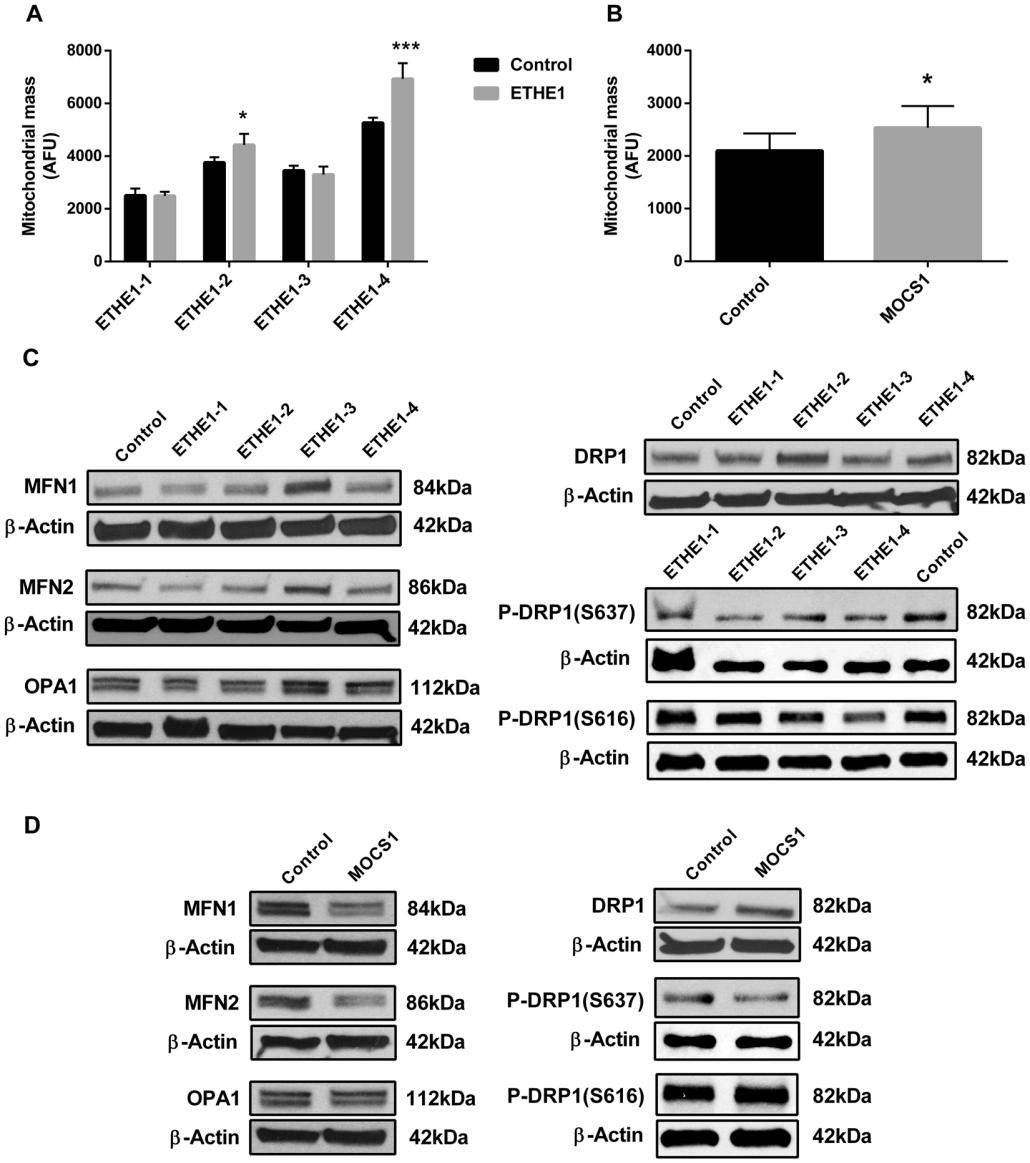
Disturbed mitochondrial mass and dynamics in ETHE1 and MOCS1 deficient fibroblasts. To measure mitochondrial mass, fibroblasts were incubated with MitoTracker Green (**A**,**B**). Data are presented as mean ± SD; number of replicates: 4–5. *P < 0.05, ***P < 0.001, compared to normal control (wild type) cells (*t* test for unpaired samples). As surrogates for mitochondrial dynamics, mitofusin 1 (MFN1), mitofusin 2 (MFN2), optic atrophy type 1 (OPA1), dynamin-related protein 1 (DRP1), p-DRP1 (S637) and p-DRP1 (S616) protein content was evaluated in whole cell lysates prepared from ETHE1 and MOCS1 deficient cells (**C**,**D**). β-actin was used as loading control. Representative images are shown.

Mitochondrial mass is often increased in the face of respiratory chain dysfunction^40,43,44^, and so this parameter was evaluated in patient cells using the probe MitoTracker Green (Fig. 5A,B), a mitochondrial dye that localizes to mitochondria with minimal dependence on mitochondrial membrane potential. ETHE1-2, ETHE1-4 and MOCS1 deficient cells were found to have increased mitochondrial mass compared to control fibroblasts, while no differences were observed in ETHE1-1 and ETHE1-3 fibroblasts. Since alterations in mitochondrial mass might result from changes in mitochondrial dynamics, we measured the levels of the main proteins involved in mitochondrial fusion (mitofusin 1 - MFN1, mitofusin 2 - MFN2, and optic atrophy type 1 - OPA1) and fission (dynamin related protein 1 - DRP1). We also determined the phosphorylation of DRP1 on Ser637 and Ser616 as it has been reported that both total levels of DRP1 and its phosphorylation play a role in regulation of mitochondrial fission^45^. MFN1 and MFN2 content was decreased in ETHE1-1, whereas MFN1, MFN2 and OPA1 content was increased in ETHE1-3 (Fig. 5C and Supplementary Fig. S2). DRP1 was increased in ETHE1-2 (Fig. 5C and Supplementary Fig. S2). Furthermore, Ser637 phosphorylation of DRP1 was markedly decreased in all ETHE1 deficient cell lines, whereas Ser616 phosphorylation was mildly decreased in ETHE1-2, ETHE1-3 and ETHE1-4 (Fig. 5C and Supplementary Fig. S2). MOCS1 deficient cells had lower levels of MFN1 and MFN2, but no alterations in OPA1 content, compared to control cells (Fig. 5D and Supplementary Fig. S3). DRP1 content was increased and Ser637 phosphorylation of DRP1 was decreased in this cell line (Fig. 5D and Supplementary Fig. S3). Phosphorylation of Ser616 of DRP1 was not altered (Fig. 5D and Supplementary Fig. S3).

### Superoxide production

**Figure 6:**
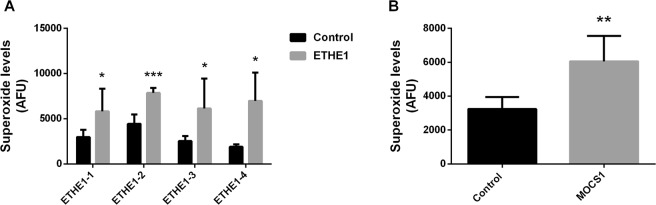
Elevated superoxide levels in ETHE1 and MOCS1 deficient fibroblasts. ETHE1 (**A**) and MOCS1 (**B**) deficient fibroblasts were incubated with MitoSOX Red. Data are presented as mean ± SD; number of replicates: 4–5. *P < 0.05, **P < 0.01, ***P < 0.001, compared to control (wild type) cells (*t* test for unpaired samples).

**Figure 7:**
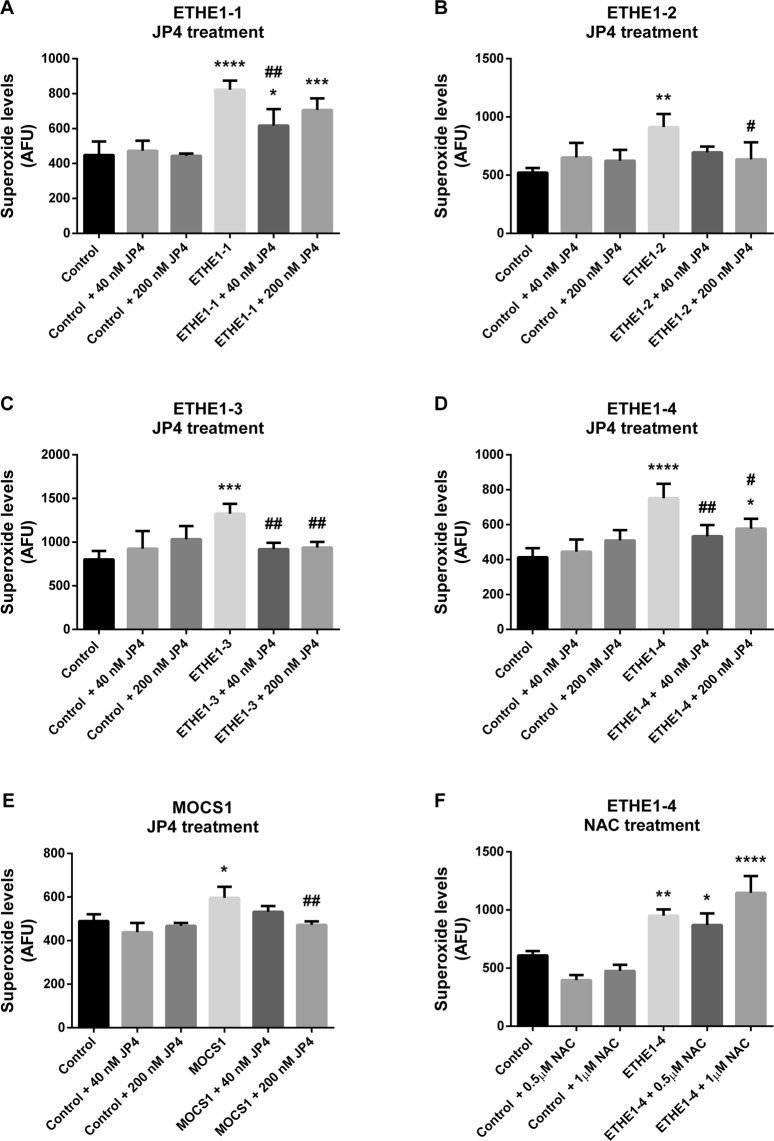
Elevated superoxide levels in ETHE1 and MOCS1 deficient fibroblasts are reduced by JP4-039. Superoxide levels were evaluated in ETHE1-1, ETHE1-2, ETHE1-3, ETHE1-4 and MOCS1 deficient fibroblasts treated with JP4-039 (JP4) (**A**–**E**) or in ETHE1-4 treated with *N*-acetylcysteine (NAC) (**F**). ETHE1 deficient cells were treated with JP4 (40 or 200 nM) or NAC (0.5 and 1 µM) during 24 h and incubated with MitoSOX Red. MOCS1 deficient fibroblasts were treated with JP4-039 (40 or 200 nM) for 7 days and incubated with MitoSOX Red. Data are presented as mean ± SD; number of replicates: 3–4. *P < 0.05, **P < 0.01, ***P < 0.001, ****P < 0.0001, compared to control (wild type) cells; ^#^*P* < 0.05, ^##^*P* < 0.01, compared to patient cells (Tukey multiple range test).

Since mitochondrial respiratory chain dysfunction is often associated with increased ROS production^40,46^, we measured superoxide levels in patients’ fibroblasts using the MitoSOX Red probe. Superoxide levels were elevated in all ETHE1 fibroblasts and the MOCS1 deficient cell line compared to control cells (Fig. 6A,B). Treatment with 40 and 200 nM JP4-039 decreased superoxide levels in MOCS1 and all of the ETHE1 deficient fibroblasts (Fig. 7A–E). In contrast, *N*-acetylcysteine, an antioxidant currently used as a therapeutic strategy for ETHE1 deficiency, had no effect on superoxide levels in ETHE1-4 (Fig. 7F).

### Protein content of ER-mitochondria crosstalk

**Figure 8:**
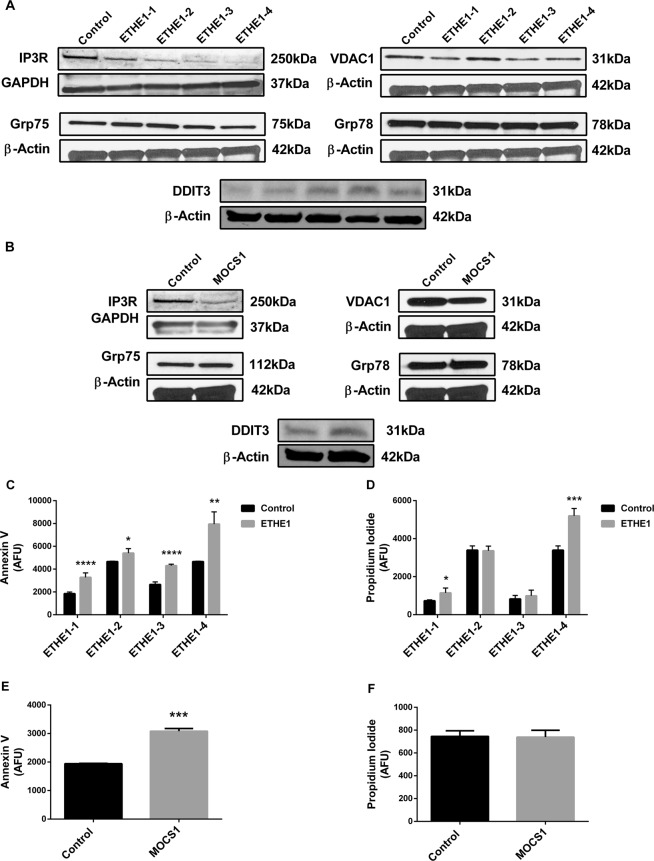
Disruption of endoplasmic reticulum-mitochondria crosstalk and apoptosis in ETHE1 and MOCS1 deficient fibroblasts. Inositol 1,4,5-trisphosphate receptor (IP3R), glucose-related protein 75 (Grp75), voltage-dependent anion-selective channel 1 (VDAC1), glucose-related protein 78 (Grp78) and DNA damage inducible transcript 3 (DDIT3) protein content was evaluated in whole cell lysates prepared from ETHE1 (**A**) and MOCS1 (**B**) deficient fibroblasts. β-Actin or GAPDH was used as loading controls. Representative images are shown. Apoptosis and necrosis in ETHE1 (**C**,**D**) and MOCS1 (**E**,**F**) deficient fibroblasts were measured by flow cytometry. Data are presented as mean ± SD; number of replicates: 3–5. *P < 0.05, **P < 0.01, ***P < 0.001, ****P < 0.0001, compared to control (wild type) (*t* test for unpaired samples).

Changes in ER-mitochondria crosstalk and ER stress are intimately related to mitochondrial dysfunction and increased ROS production^47,48^. Therefore, we evaluated the content of proteins involved in ER-mitochondria crosstalk (voltage-dependent anion-selective channel 1 - VDAC1, inositol 1,4,5-trisphosphate receptor - IP3R, and glucose-related protein 75 - Grp75) and ER stress (DNA damage inducible transcript 3 - DDIT3, and glucose-related protein 78 - Grp78) in the fibroblasts from ETHE1 and MOCS1 deficient patients. All ETHE1 deficient cell lines showed decreased IP3R and increased DDIT3 content, whereas VDAC1 was decreased in ETHE1-1, ETHE1-3 and ETHE1-4 fibroblasts (Fig. 8A and Supplementary Fig. S4). IP3R and VDAC1 were also decreased, and DDIT3 was increased in MOCS1 deficient fibroblasts (Fig. 8B and Supplementary Fig. S5). No alterations were observed in Grp75 and Grp78 in any cell line evaluated.

### Apoptosis

Cell death was evaluated by assay of annexin V (early apoptosis) and propidium iodide (PI) (late apoptosis and necrosis). Early stage apoptosis was increased in all patient cell lines (Fig. 8C,E, Supplementary Figs S6 and S7), whereas late stage apoptosis and cell necrosis were increased only in ETHE1-1 and ETHE1-4 fibroblasts (Fig. 8D and Supplementary Fig. S6).

## Discussion

ETHE1 and MoCo deficiencies are devastating inborn errors in the metabolism of sulfur-containing amino acids. While respiratory chain dysfunction has been demonstrated and increased oxidative stress has been postulated, the precise mechanism of cellular pathophysiology remains unclear, and therapies remain largely ineffective. The aim of this study was to further examine cellular derangements in ETHE1 and MoCo deficient fibroblasts and evaluate their response to a novel mitochondrial antioxidant. After confirming the expected mutations in each cell line, we examined a series of parameters of mitochondrial bioenergetics and homeostasis. All of the patient fibroblasts demonstrated decreased mitochondrial respiration. This could be observed despite that previous studies showed reversion of H_2_S-induced inhibition of cytochrome *c* oxidase activity in ETHE1 deficient fibroblasts when exposed to air^14,49^. Furthermore, a recent article on ETHE1 deficient fibroblasts did not show impairment in mitochondrial respiration^50^. This discrepancy might be because they analyzed cells that had been adhering for more than a day whereas we seeded the cells in microplates pre-coated with poly-D-lysine and measured the respiration directly. On the other hand, this same study described a growth initiation phenotype of ETHE1 deficient cells^50^, which is in line with our data on respiration defect of freshly adhered cells. The decreased respiration found here is further in line with data showing cytochrome *c* oxidase depletion in *Ethe1*^−/−^ mice, and impairment of mitochondrial energetic homeostasis caused by metabolites accumulating in ETHE1 deficiency and MoCD in rat tissues^15,27,28,51^. Additionally, MOCS1 and ETHE1 deficient cells showed reduced ATP production, confirming that bioenergetics is compromised in these cells.

Mitochondria are highly dynamic organelles that are constantly undergoing fusion and fission, controlling their shape and playing a fundamental role in the adaptation to disturbances in the cellular environment^52–54^. Changes in the cell bioenergetic state usually alter mitochondrial morphology and dynamics, leading to dysfunction^55^. We therefore investigated the content of the main proteins involved in mitochondrial dynamics, namely MFN1, MFN2, OPA1 (fusion) and DRP1 (fission). ETHE1-3 cells showed higher levels of all fusion proteins evaluated, while ETHE1-2 had increased levels of DRP1. Phosphorylation of DRP1 Ser637 and Ser616 was also determined as these post-translational modifications regulate DRP1-mediated fission. In this context, phosphorylation of Ser616 promotes Drp1-dependent mitochondrial fission, whereas Ser637 phosphorylation suppresses fission through the inhibition of the intramolecular interaction between GTPase and GED domains, thereby hampering the DRP1 GTPase activity and its recruitment to mitochondria^56,57^. While Ser637-phosphorylated DRP1 levels were markedly decreased in all cell lines, Ser616-phosphorylated DRP1 was mildly decreased in ETHE1-2, ETHE1-3 and ETHE1-4 deficient cells. Mitochondrial mass was further evaluated and an increase in this parameter was found in ETHE1-2 and ETHE1-4 cells. These changes suggest a variety of responses to maintain cellular energy homeostasis and reduce ROS levels^58–61^. MOCS1 deficient cells had a reduction in MFN1 and MFN2, an increase in DRP1, a decrease in Ser637-phosphorylated DRP1 and enhanced mitochondrial mass, indicating severe mitochondrial dysfunction and an increased number of smaller mitochondria.

The alterations in mitochondrial bioenergetics and dynamics were accompanied by increased superoxide production in all cell lines studied, the first direct demonstration of this finding, and consistent with previous data showing an abnormal proteome and decreased levels of reduced glutathione in ETHE1 deficiency cells, indicating disruption of redox homeostasis^30,50^. Moreover, accumulation of H_2_S and sulfite in induced ETHE1 and MoCo deficiency, respectively, elicits an oxidative stress response in rat brain^27,28,51,62^. Mitochondria are the main source of superoxide generation, predominantly in the redox centers of the respiratory chain complexes I and III from which electrons may leak during oxidative phosphorylation, leading to the incomplete reduction of oxygen. The physiological production of ROS by these sites plays an important role in cellular signaling, but the exacerbation of this process has been implicated in several diseases^63–67^ since ROS may cause structural damage that exceeds cellular repair capacity, triggering cellular dysfunction^63^. Additionally, production of ROS induced by other cellular sources or accumulated metabolites may also result in secondary impairment of the respiratory chain components with further increase of superoxide production by mitochondria^68–70^.

Mitochondrial bioenergetic dysfunction and oxidative stress are associated with disruption of ER-mitochondria communication and ER stress^47,71–73^. Crosstalk between mitochondria and ER is crucial for the regulation of mitochondrial dynamics and the signaling of calcium in cells, and is accomplished through the interaction of the VDAC1 in mitochondria and the IP3R in the ER, which are anchored by Grp75^74–76^. In our ETHE1 and MOCS1 deficient cells, a reduced content of VDAC1 and IP3R was found, implying a disturbance in the ER-mitochondria crosstalk. Furthermore, DDIT3, an ER stress marker, was increased in all cell lines. The upregulation of DDIT3 is consistent with an increase in apoptosis induction observed in these cells, since this transcription factor is known to modulate this process^77^.

Since increased ROS production contributes to the damage of a series of biomolecules, including respiratory chain components^68–70^, it is reasonable to expect that the mitigation of ROS production would be of therapeutic value in ETHE1 and MoCo deficiencies. We have previously demonstrated that treatment with the novel mitochondria-targeted antioxidant and electron scavenger JP4-039, reduces ROS levels in cells with impairments in oxidative phosphorylation, while other clinically used antioxidants did not^40^. In the current study, we also verify that JP4-039 ameliorates ROS accumulation in ETHE1 and MOCS1 deficient fibroblasts and improves mitochondrial respiration. These findings are particularly interesting since previous data showed that JP4-039 crosses brain-blood barrier in mice^78^ and ETHE1 and MOCS1 deficiencies are inborn errors mainly characterized by neurological dysfunction. Furthermore, *N*-acetylcysteine, an antioxidant currently used to treat ETHE1 deficient patients^10,13,79,80^, did not affect superoxide levels, demonstrating the importance of mitochondrial targeting for the biological efficacy of free radical scavengers.

Interestingly, the different ETHE1 deficient fibroblast cell lines showed some heterogenous alterations that could be at least partially explained by the different mutations. However, even different cell lines with the same mutation (ETHE1-1 and ETHE1-4) showed some variability in mitochondrial function, consistent with previous conclusions that there is no clear genotype-phenotype correlation in this disease^80–82^. The reasons for these variations and their correlation with clinical symptoms remain to be clarified, and may ultimately be useful for the identification of additional specific therapies. Furthermore, it is difficult to extrapolate the pathophysiological relevance of fibroblast findings to other tissues, though, in general, it is harder to demonstrate bioenergetic dysfunction in fibroblasts than in more mitochondria-rich tissues. Thus, it is likely that our fibroblast findings are relevant to tissues with higher energetic needs.

## Conclusions

In summary, we have demonstrated that fibroblasts from patients with ETHE1 deficiency and MoCD have impairments in mitochondrial bioenergetics and dynamics, as well as disruption of ER-mitochondria crosstalk. High superoxide levels identified in these cells presumably play an important role in the cellular pathophysiology, which is improved by treatment with the mitochondrial-targeted ROS and electron scavenger JP4-039. Thus, this compound is an interesting candidate for further studies as a novel therapeutic agent for treatment of ETHE1 deficiency and MoCD.

## Supplementary information

Supplementary data

## Data Availability

The datasets supporting the conclusions of this article are included within the article and its additional file.
