# Supplementary material for: ETHE1 and MOCS1 deficiencies: Disruption of mitochondrial bioenergetics, dynamics, redox homeostasis and endoplasmic reticulum-mitochondria crosstalk in patient fibroblasts
Source: Sci Rep. 2019 Sep 2;9:12651. doi: 10.1038/s41598-019-49014-2 (PMC6718683; doi:10.1038/s41598-019-49014-2)
Supplement: Supplementary file 1 — Supplementary data [file 41598_2019_49014_MOESM1_ESM.doc]

**SUPPLEMENTARY INFORMATION**

**Title of the manuscript:** ETHE1 and MOCS1 deficiencies: Disruption of mitochondrial bioenergetics, dynamics, redox homeostasis and endoplasmic reticulum-mitochondria crosstalk in patient fibroblasts

**Authors:** Mateus Grings, Bianca Seminotti, Anuradha Karunanidhi, Lina Ghaloul-Gonzalez, Al-Walid Mohsen, Peter Wipf, Johan Palmfeldt, Jerry Vockley, Guilhian Leipnitz

**Table S1.** **Genomic DNA mutations of patients with molybdenum cofactor and ETHE1 deficiencies**

| **Patients** | **Mutated Gene** | **Genotype** |
| --- | --- | --- |
| **MoCD** | *MOCS1* | c. 199C>T; p.Arg67Trp  c.377G>A; p.Gly126Asp |
| **ETHE1-1** | *ETHE1* | Exon 4 deletion |
| **ETHE1-2** | *ETHE1* | c.494A>G; p.Asp165Gly |
| **ETHE1-3** | *ETHE1* | c.505 +1 G>T |
| **ETHE1-4** | *ETHE1* | Exon 4 deletion |

Mutations were determined in fibroblasts of patients with molybdenum cofactor deficiency (MoCD) and ethylmalonic encephalopathy protein 1 (ETHE1) deficiency. All mutations were homozygous except for *MOCS1* gene where the patient was compound heterozygous for 2 mutations.





**Figure S1. ATP-linked respiration decrease in ETHE1 and MOCS1 deficient fibroblasts.** Oxygen consumption rate (OCR) was measured after injection of oligomycin (an inhibitor of ATP synthase), representing ATP-linked respiration turnover. ATP-linked respiration of ETHE1-1 (A), ETHE1-2 (B), ETHE1-3 (C), ETHE1-4 (D) and MOCS1 (E) deficient fibroblasts exposed or not to 40 nM JP4-039 (JP4) for 24 h. Data are means ± SD; number of replicates: 7-8. **P*<0.05, ***P*<0.01, ****P*<0.001, *****P*<0.0001, compared to control cells; ^#^*P*<0.05, ^##^*P*<0.01, ^###^*P*<0.01, compared to patient cells (Tukey multiple range test).

**

Figure S2.** **Representative histograms of mitochondrial dynamics protein content in ETHE1 deficient fibroblasts**. The content of the mitochondrial dynamics proteins mitofusin 1 (MFN1), mitofusin 2 (MFN2), optic atrophy type 1 (OPA1), dynamin-related protein 1 (DRP1), p-DRP1 (S637) and p-DRP1 (S616) was evaluated by western blotting in whole cell lysates prepared from ETHE1 deficient cells. β-actin was used as loading control.

**

Figure S3. Representative histograms of mitochondrial dynamics protein content in MOCS1 deficient fibroblasts**. The content of the mitochondrial dynamics proteins mitofusin 1 (MFN1), mitofusin 2 (MFN2), optic atrophy type 1 (OPA1), dynamin-related protein 1 (DRP1), p-DRP1 (S637) and p-DRP1 (S616) was evaluated by western blotting in whole cell lysates prepared from MOCS1 deficient cells. β-actin was used as loading control.

**

**

**Figure S4. Representative histograms of endoplasmic reticulum-mitochondria crosstalk protein content in ETHE1 deficient fibroblasts**. The content of the endoplasmic reticulum-mitochondria crosstalk proteins inositol 1,4,5-trisphosphate receptor 3 (IP3R), glucose-related protein 75 (Grp75), voltage-dependent anion-selective channel 1 (VDAC1), glucose-related protein 78 (Grp78) and DNA damage inducible transcript 3 (DDIT3) was evaluated by western blotting in whole cell lysates prepared from ETHE1 deficient cells. β-Actin or GAPDH were used as loading controls.

**

Figure S5. Representative histograms of endoplasmic reticulum-mitochondria crosstalk proteins content in MOCS1 deficient fibroblasts**. The content of the endoplasmic reticulum-mitochondria crosstalk proteins inositol 1,4,5-trisphosphate receptor (IP3R), glucose-related protein 75 (Grp75), voltage-dependent anion-selective channel 1 (VDAC1), glucose-related protein 78 (Grp78) and DNA damage inducible transcript 3 (DDIT3) was evaluated by western blotting in whole cell lysates prepared from MOCS1 deficient cells. β-Actin or GAPDH were used as loading controls.


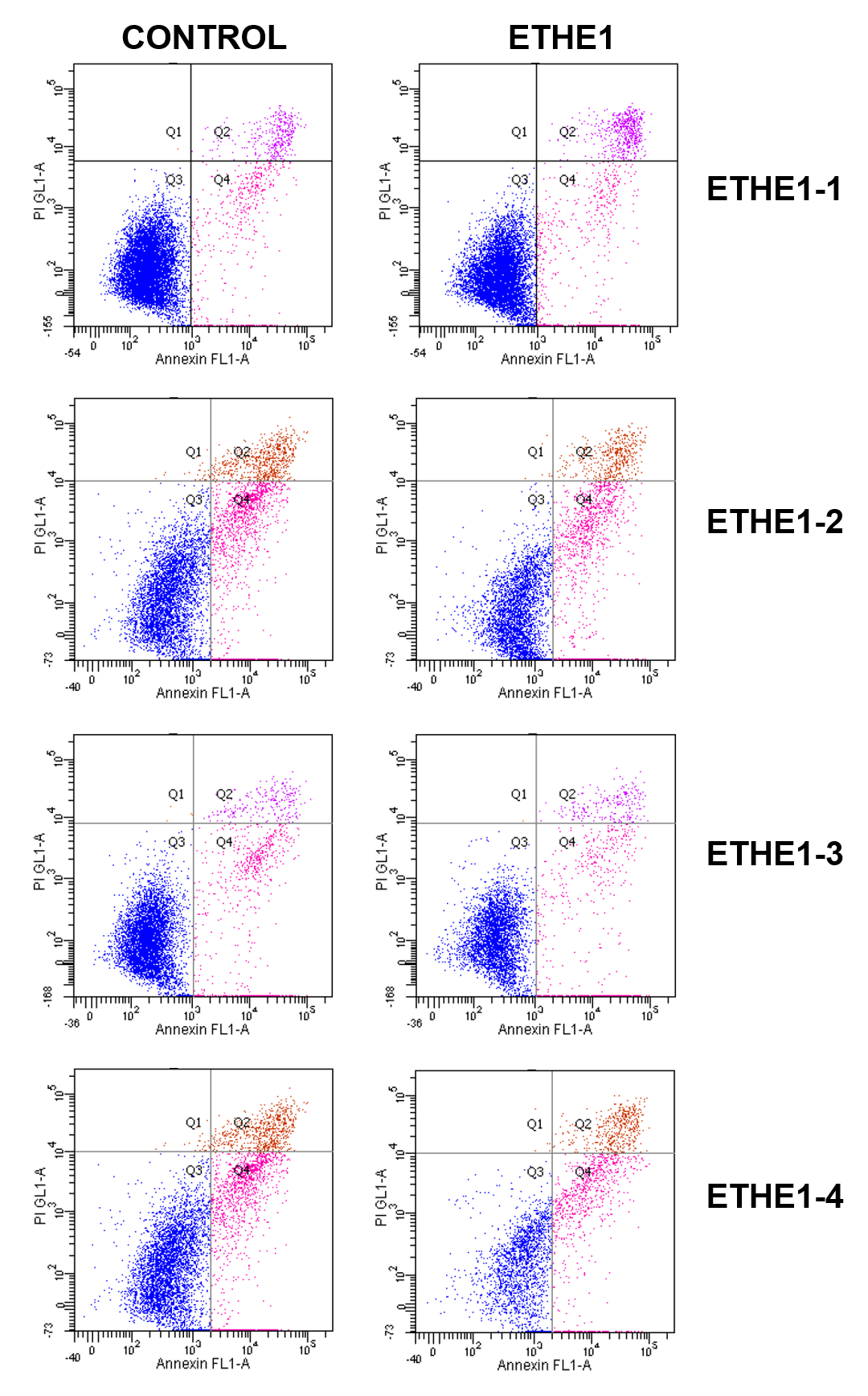


**Figure S6. Representative dot plots of control and ETHE1 deficient fibroblasts (Annexin V versus Propidium iodide) analyzed by flow cytometry.**

**
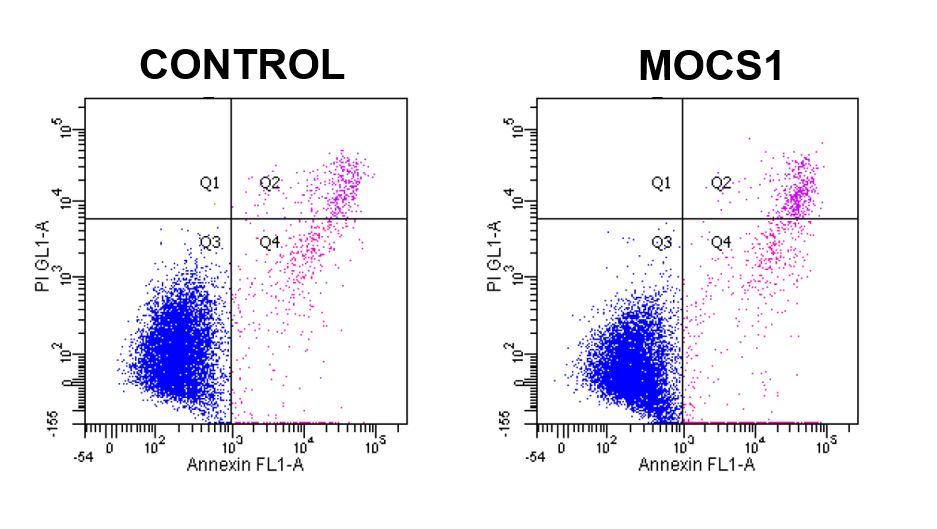
**

**Figure S7. Representative dot plots of control and MOCS1 deficient fibroblasts (Annexin V versus Propidium iodide) analyzed by flow cytometry.**
